# On Abdominal Pressure in Obstetrics

**Published:** 1831-01-01

**Authors:** 


ABDOMINAL PRESSURE IN OBSTETRICS.

MR. M'MORRIS, a medical officer in the East India Company's service, writes to us as follows. "In one of the numbers of the Medico-chirurgical Review for 1828, I find a bandage proposed by Mr. Gaitskell for obstetric purposes. I have no doubt it will facilitate the parturient powers of Nature by the support and pressure which it affords. It is a common auxiliary among the native women on this side of the Indian peninsula, when the progress of labour is slow. Some of the female attendants, in such cases, place their hands on the abdomen of the parturient patient, and press it pretty violently downwards towards the pubes, having first rubbed it with castor oil. This operation is continued till the child is expelled, which generally happens soon after the mechanical pressure is commenced. If the secundines are not expelled with the fœtus, the woman is left to rest, with a bandage applied pretty tightly round the abdomen. I have been astonished, in some cases that I have witnessed, to observe the increase of the expulsive pains soon after this process is put in force."—*Delhi*, 1830.

N.B. The above procedure corresponds with that recommended a few years ago by Dr. Power, and which was, at the time, too slightingly entertained by our obstetric brethren.—*Ed.*

